# GMDD: a database of GMO detection methods

**DOI:** 10.1186/1471-2105-9-260

**Published:** 2008-06-04

**Authors:** Wei Dong, Litao Yang, Kailin Shen, Banghyun Kim, Gijs A Kleter, Hans JP Marvin, Rong Guo, Wanqi Liang, Dabing Zhang

**Affiliations:** 1 GMO Detection Laboratory, SJTU-Bor Luh Food Safety Center, Key Laboratory of Microbial Metabolism, Ministry of Education, School of Life Science and Biotechnology, Shanghai Jiao Tong University, 800 Dongchuan Road, Shanghai 200240, PR China; 2 Shanghai Fisheries University, 334 Jungong Road., Shanghai 200090, PR China; 3 Korea Food & Drug Administration, 194 Tongiliro, Eunpyung-gu, Seoul 122-704, South Korea; 4 RIKILT – Institute of Food Safety, Wageningen University and Research Center, Bornsesteeg 45, NL-6708 PD Wageningen, The Netherlands; 5 Shanghai University, 149 Yanchang Road., Shanghai 200072, PR China

## Abstract

**Background:**

Since more than one hundred events of genetically modified organisms (GMOs) have been developed and approved for commercialization in global area, the GMO analysis methods are essential for the enforcement of GMO labelling regulations. Protein and nucleic acid-based detection techniques have been developed and utilized for GMOs identification and quantification. However, the information for harmonization and standardization of GMO analysis methods at global level is needed.

**Results:**

GMO Detection method Database (GMDD) has collected almost all the previous developed and reported GMOs detection methods, which have been grouped by different strategies (screen-, gene-, construct-, and event-specific), and also provide a user-friendly search service of the detection methods by GMO event name, exogenous gene, or protein information, etc. In this database, users can obtain the sequences of exogenous integration, which will facilitate PCR primers and probes design. Also the information on endogenous genes, certified reference materials, reference molecules, and the validation status of developed methods is included in this database. Furthermore, registered users can also submit new detection methods and sequences to this database, and the newly submitted information will be released soon after being checked.

**Conclusion:**

GMDD contains comprehensive information of GMO detection methods. The database will make the GMOs analysis much easier.

## Background

With the development of modern biotechnology, numerous genetically modified organisms (GMOs) have been approved for commercialization. However, GMOs' emergence has caused public debate on the consumers' freedom of choice to purchase GMO-derived products or not. To protect the consumers' freedom of choice, more than 40 countries have issued GMO labelling regulations, for instance, GM foods must be labelled at the threshold of 0.9% in European Union (EU), 3% in Korea, and 5% in Japan [1-3]. In China, no matter how small the GM content is, 17 types of GM products must be labelled, such as maize seeds, maize oil, tomato seeds, ketchup, soybean seeds, soybean oil, rapeseed seeds, and cotton seeds [4].

To make sure the successful enforcement of the GMO labelling, protein and nucleic acid-based detection techniques have been developed, or are in the process of development, such as ELISA, lateral flow strip, PCR, and micro-array etc [5-13]. Among these techniques, PCR is the most popular technique for GMOs analysis because of its versatility (from screening to identification), specificity, high throughput, and efficiency, therefore the PCR detection methods have been developed for many GMOs. The introduced DNA fragments are usually integrated into the genome of GMOs by random transformation events: based on the different amplification fragments of the inserted DNA sequences, PCR detection methods of GMOs are grouped into four types, such as the screen-, gene-, construct-, and event-specific PCR methods [6,7]. Moreover, to overcome the difficulties in obtaining the certified reference materials (CRMs) of GMOs, standard reference molecules are developed and used in PCR detection [14-16]. This is a new research hotspot in the field of GMO detection, and this will greatly facilitate the utilization of PCR detection methods.

Up to now, hundreds of GMOs detection methods have been developed, and their number is increasing more and more rapidly. However, with so many detection methods to choose, an analyst may feel confused. To select the most informative analyses beforehand, it is necessary to establish one public database for GMOs analysis methods, providing update analysis information to the analysts. Even though there are some GMO databases related to GMO safety assessment, including Agbios [17], GMO-Compass [18], and Biodiv LMO Database [19], etc (all the databases are available till now, 17^th ^April, 2008), these databases are mainly engaged in collecting the information of GMOs' risk assessment, and relatively little information on GMO detection is included.

Here, we report the development of a new database for GMO detection methods. In this database, we have collected almost all the previous developed GMOs detection methods, thus providing a user-friendly search service for GMOs by event name, gene, and protein information, etc. In particular, we supply sequence information of exogenous inserts, if available, as well as endogenous reference genes, and standard reference materials for GMOs analysis in our database. Furthermore, registered users can submit new GMO detection methods or sequences to this database, which makes this database open.

## Construction and content

### System architecture

The whole system is developed with PHP5 (using framework for development, including Zend Framework 1.0 and Smarty 2.6), MySQL5 for primer information storage. Besides, NCBI BLAST2, and BioPerl package [20] are also introduced to GMDD to achieve a BLAST function for all the exogenous inserted sequences. WWW Primer 3 [21] is also integrated into GMDD, which will help users with primers' design.

### Data collection

In this GMO detection method database (GMDD), we provide the information on two aspects of each approved GMO event, one is the general information on each transformation event for detection purposes, and the other is its detailed detection method information. The general information of GMO events includes the event name, OECD UI (Organization for Economic Co-operation and Development OECD Unique Identifier), trade name, species, newly introduced traits, exogenous inserts, transformation methods, transformation vectors and developing or licensed company.

In particular, the inserted sequences of GMOs in GMDD have been collected from published research articles, Genbank [22], patents, and producer's petitions to governments, some sequences are assembled by Vector NTI Advance™ 10.3 Contig Express software [23]. In addition to the sequence information, for other information on the transformation event, references have been cited from the original US petitions, Japanese petitions, Agbios [17], GMO-Compass [18], Biodiv LMO Database [19], and GMO watch report [24], etc.

The GMOs detection method information is grouped into nucleic acid-based method and protein-based method. The nucleic acid-based method information includes primers, probes, amplicon length, figures showing primer pair positions, endogenous reference genes, certified reference materials, standard reference molecules, information on validation status, and reference articles. Currently, most protein-based methods are based on commercialized kits, and that is the reason why GMDD covers both commercial kits as well as non-kits methods. The GMO detection information in GMDD has been mainly collected from published research papers, Community Reference Laboratory for Genetically Modified Food and Feed (CRL-GMFF) validation reports, national standards and international standards, for instance, China and international standardization organization (ISO) standards, etc.

### Data quantity

By mid April 2008, we have collected a total number of 136 GMO events, more than 400 pairs of PCR detection primers, 30 protein-based methods, 43 endogenous reference genes from 17 taxa, 91 certified reference materials, 9 standard reference molecules, and more than 44 inserted sequences in GMDD.

## Utility and discussion

### Data retrieval

**Figure 1:**
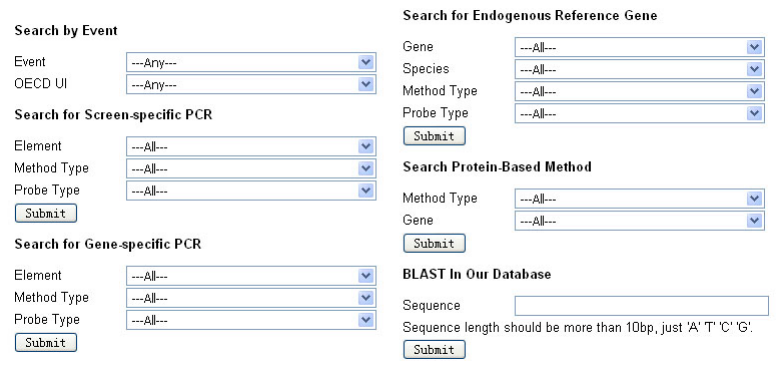
**GMDD search interface**. i) Users can search the information by event information, ii) or by detection method information, iii) or search inserted sequences by a BLAST function.

The GMO detection method information can be searched directly by full event name and by OECD unique identifier. For methods which can be used for testing more than one GMO event (such as screen-, gene-specific PCR, protein-based methods), users can access them by the methods' own search options. For example, gene-specific PCR methods can be searched by their target gene as well as by primer/probe type. For the exogenous DNA sequences of GMOs, users can obtain the available information via the transformation event name or via the BLAST function as provided by GMDD (Figure 1).

### Data listed by transformation event

**Figure 2:**
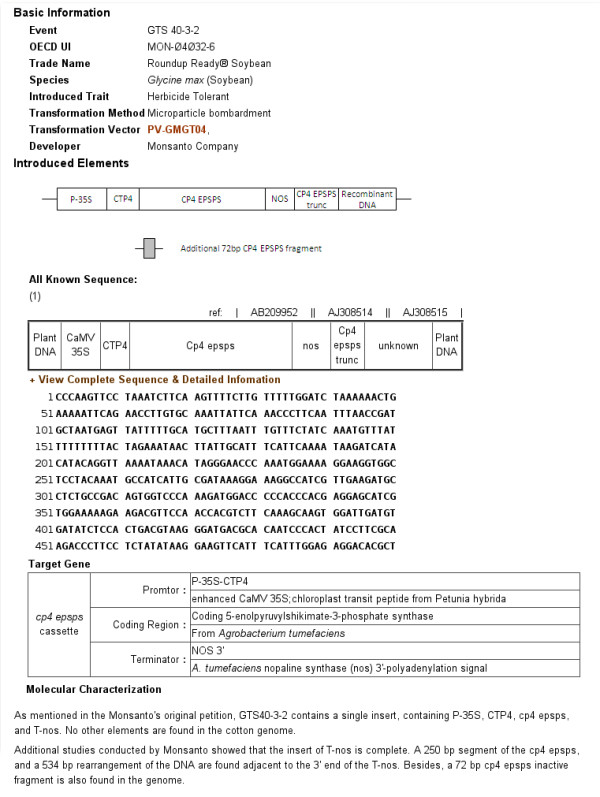
**Basic information of GTS 40-3-2**. Information is grouped into i) basic information, ii) introduced elements: including general figure, sequences (if the sequence was longer than 500 bp, only the first 500 bp would be displayed on this page), gene elements illustration, and molecular characterization.

**Figure 3:**
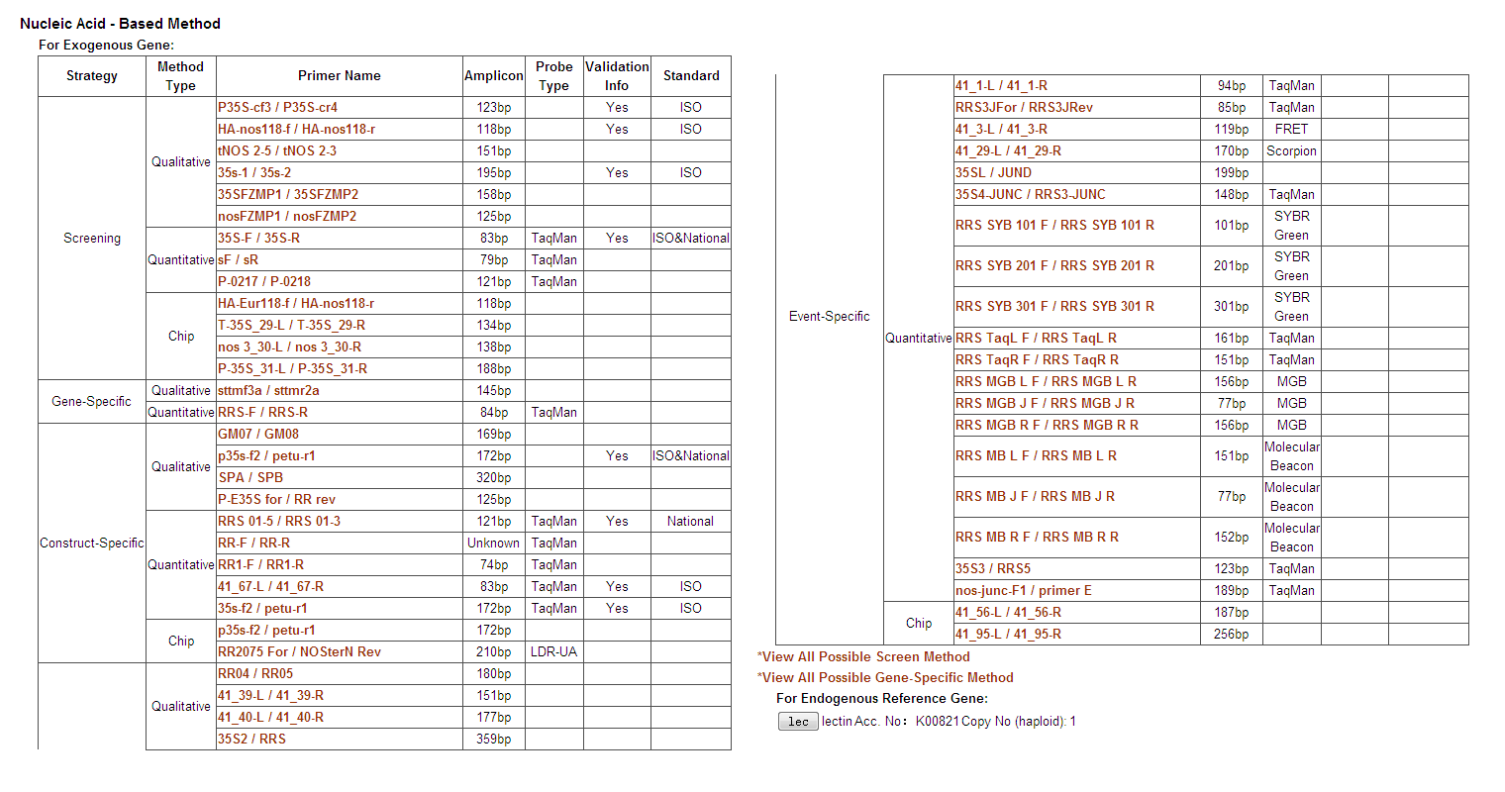
**Nucleic acid-based method of GTS 40-3-2**. i) Primers information is grouped into 4 different PCR strategies, screen-, gene-specific, construct-specific, and event-specific, ii) Predicted possible primers, iii) Endogenous reference gene, iv) Standard reference molecules.

**Figure 4:**
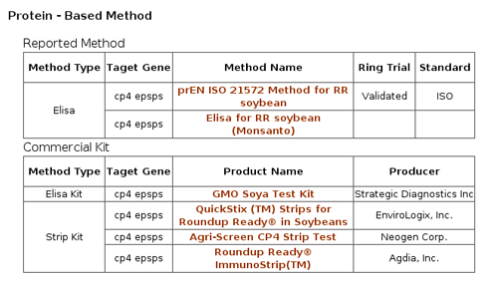
**Protein-based detection methods of GTS 40-3-2**. These are classified into reported method and commercial kit.

**Figure 5:**
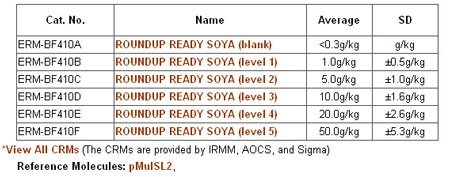
Certified reference materials of GTS 40-3-2.

The general information and analysis data of each GMO in GMDD are presented in the following five main sections: basic information, insertion elements, nucleic acid-based method, protein-based method, and reference materials (Figure 2, Figure 3, Figure 4 and Figure 5). The basic information on each GMO in GMDD comprises the following data, i.e. event name, OECD Unique Identifier, trade name, species, GM new trait, gene introduction method, transformation vector, developing and/or trading company. The information on insertion elements includes a general figure of the inserted elements, the known part of the inserted sequences with sketch maps, and the detailed information on gene elements. With this information, users may design new primers or probes more easily, or confirm their detection results with the provided sequences (Figure 2).

The nucleic acid-based methods include the information of PCR primers, probes, endogenous reference gene, and information on the validation status of the method. PCR analysis information is generally grouped into 4 different PCR strategies, screen-, gene-, construct-, and event-specific. Each group is classified into quantitative and qualitative amplification method including further hybridization on chip. For each gene element, all the relevant screen- and gene-specific PCR primers are listed (the information could be retrieved by clicking "View All Possible Screen Method" or "View All Possible Gene-Specific Method"). Furthermore, there is a list of all known endogenous reference genes by taxon. By clicking on the gene name, users will get a list of primers for this gene (Figure 3).

Protein-based methods (including both kits and non-kits methods) cover both ELISA and lateral flow strip methods, and also the information on the validation status of the methods and whether standards for non-kits methods are provided (Figure 4).

In the certified reference materials section, users may find out such information as CRM name, catalogue number, composition and measured content with their measurement uncertainty. The information on standard reference molecules is also included in the certified reference materials section. Users can get detailed information by clicking on the name of standard reference molecules (Figure 5).

### Detailed method information

**Figure 6:**
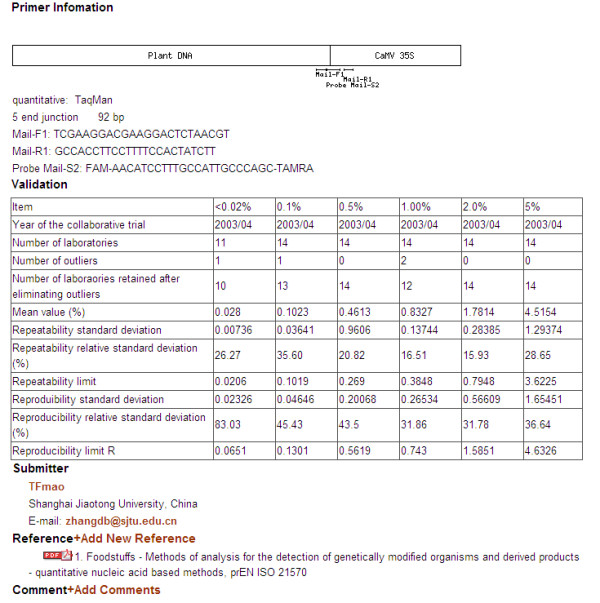
**Detailed information of GMOs analysis method**. i) Target event information, ii) primer/probe information and a figure indicating the position where primers pair with the exogenous elements, iii) validation information, iv) submitter information, v) reference and comment, registered users can add references and comments by clicking on the hypertext link.

By clicking on the hypertext link of the primers list, users can get access to the detailed method information. Nucleic acid-based methods' information includes the type of analysis (quantitative, qualitative amplification methods including further hybridization on chip), primer/probe type (TaqMan^®^, Molecular Beacon, and FRET, etc), primer/probe name, primer/probe sequence, amplicon length, information on validation status, references, standard information, and data on the source of the information. In addition, there are PCR reaction parameters for some of the validated detection methods. For screen- and gene-specific method, there is also a list of transformation event names validated by research articles. For construct- and event-specific method, there is a figure indicating the primer pairing positions with the inserted elements, according to the information on GMO inserted sequences and primer sequences (Figure 6). Protein-based method information includes method type, method name, a list of transformation event names validated by research articles, references, and data on the source of the information.

### Submit new information to GMDD

GMDD also provides the service for users to submit new detection methods, inserted sequences and to comment or to submit supplementary information for specific methods of detection. This should be helpful for improving the information content in the future and also it will constitute a platform for information exchange between researchers and analysts in the field of GMO detection.

We encourage users to submit validated information, that is, the information should have already been published in scientific research papers. The submitted information can not be obtained by others until our administrators have checked it within about 1–2 weeks. During this period, the submitters can edit the method information, and even after we publish the new method on the website, the submitter still has full access to the methods data.

On the page of the detailed method and sequence information, users can submit supplementary references or comments (Figure 6). And if the method can be used in the detection of more than one GM event, users can also add new event names to the database. Except for the users' comments, all the other information will be checked by the administrator.

## Conclusion

With the comprehensive information of nearly all the existing GMO detection methods, including GMO basic information, transformation information and clearly classified detection methods information, and with the new method submission and comment function, GMDD will be a platform for analysts to exchange their data, issues and ideas on GMO detection. This will make the development and validation of new detection methods much easier. It will be more convenient for GMO detection laboratories choosing fit for purposes detection methods.

Because most of the GMO sequences are still unknown to us, and the sequence information contained by the GMDD database is of great importance to the development of new methods, the GMDD consortium plans further to organize a project of sequencing GMO insert border sequences or even the whole inserted sequences, alternatively, initiate the cooperation with the developing and licensed companies for retrieving more sequence information.

The GMDD consortium will also continue its cooperation with its European partner, RIKILT – Institute of Food Safety (part of Wageningen University and Research Center), by setting up a supplementary database. This joint database will particularly focus on the risk assessments that have been carried out for food and feed use of the transgenic events described by GMDD. The data thus provided by GMDD will constitute a unique combination of sequence data, detection methods, and safety data, thereby providing a useful tool for analytical method developers, regulatory officials from both industry and governments, risk assessors, risk managers, and other interested parties.

## Availability and Requirements

Home page: GMDD can be accessed at [25]

Operating systems: platform-independent

Programming language: PHP, Perl

License: Academic Free License (AFL) v. 3.0

## Abbreviations

CRL-GMFF: Community Reference Laboratory for Genetically Modified Food and Feed; CRM: Certified Reference Material; FRET: Fluorescence Resonance Energy Transfer; GMDD: GMO Detection method Database; GMO: Genetically Modified Organism; ISO: International Standardization Organization; LMO: Living Modified Organism; OECD UI: Organization for Economic Co-operation and Development (OECD) Unique Identifier

## Competing interests

All authors are professionals employed by Shanghai Jiao Tong University, Shanghai Fisheries University, Korea Food & Drug Administration, RIKILT – Institute of Food Safety, and Shanghai University, and as such do not have any interests that may conflict with the contents of the article above.

## Authors' contributions

Designing of the database: DZ, LY, WL and WD. Data collection: WD, BK (responsible for data related to Korea), GAK (responsible for data related to Europe), HJPM (responsible for data related to Europe) and LY. Data proofreading: KS. The whole programming work and web server maintenance: WDSoftware testing: RG. All authors have read and approved the final manuscript.
